# Renal carcinoid tumor with liver metastasis followed up postoperatively for 9 years

**DOI:** 10.1186/s13000-015-0417-7

**Published:** 2015-10-06

**Authors:** BinShen Ouyang, XiaoMei Ma, HongZhu Yan, Jin He, ChunYan Xia, HongYu Yu

**Affiliations:** Department of Pathology, Shanghai Chang Zheng Hospital, The Second Military Medical University, Shanghai, China

**Keywords:** Kidney, Carcinoid, Metastasis

## Abstract

**Background:**

We describe a case of renal carcinoid tumor with liver metastasis followed up postoperatively for 9 years.

**Case presentation:**

A 33-year-old man presented with left flank dull ache. On the abdominal computed tomography, a solid renal mass in the upper portion of the left kidney was detected. The patient had no other abnormal findings, such as suspected distant metastasis or lymph node metastasis. Radical nephrectomy was performed on 14/9/2005. Histological examination and immunohistochemical staining confirm primary renal carcinoid tumor. 9 years after radical nephrectomy, computed tomography of the abdomen demonstrated a 2 cm × 1.8 cm cyst mass in the right liver. Similar pathologic characteristics were found between the renal carcinoid tumor and liver tumor.

**Conclusions:**

We present a primary renal carcinoid tumor with liver metastasis 9 years after radical nephrectomy. With literature review, renal carcinoid tumors exhibit heterogenous behavior.

## Background

Carcinoid tumors of the kidney are extremely uncommon. Primary carcinoid tumors of the kidney are low-grade, malignant tumors that arise from neuroendocrine cells. Since then, no more than 100 cases have been reported in the literature, and approximately 20 cases developed liver metastases at the time of initial diagnosis [1–3]. Only two cases were found liver metastases 5 and 6 months after surgery [1, 4]. Herein, we present the case of a 33-years-old man who had primary renal carcinoid with liver metastasis followed up after radical nephrectomy for 9 years. Histological examination and immunohistochemical staining confirm consistent characteristics between liver and renal tumor.

## Case presentation

A 33-year-old man presented with left flank dull ache. On the abdominal computed tomography, a solid renal mass in the upper portion of the left kidney was identified.

**Fig. 1:**
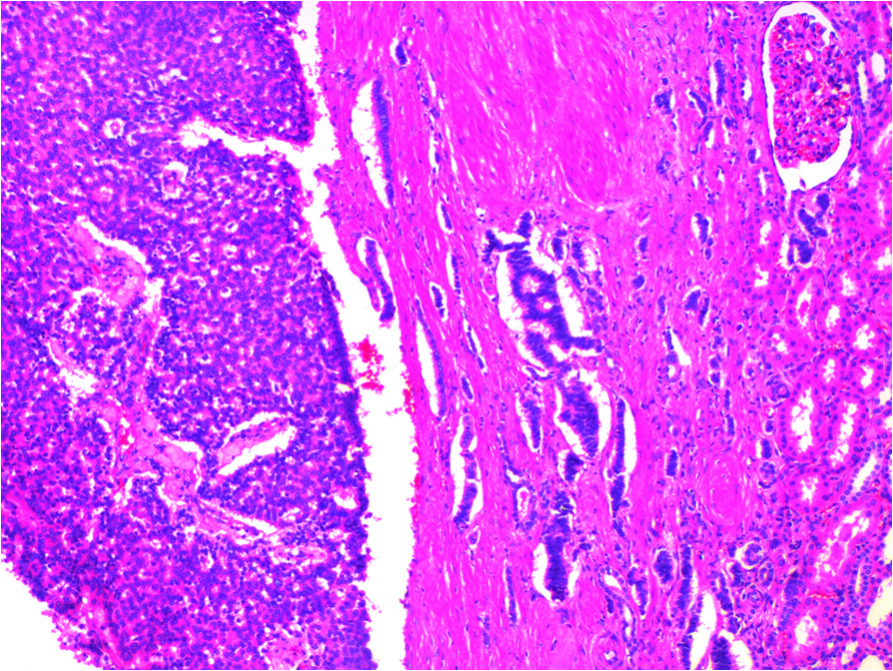
Tightly packed trabecular and glandular formations in renal carcinoid tumor with minimal stroma (left side of image), ill-defined with peripheral nephridial tissue(right side of image) (H&E × 200)

**Fig. 2:**
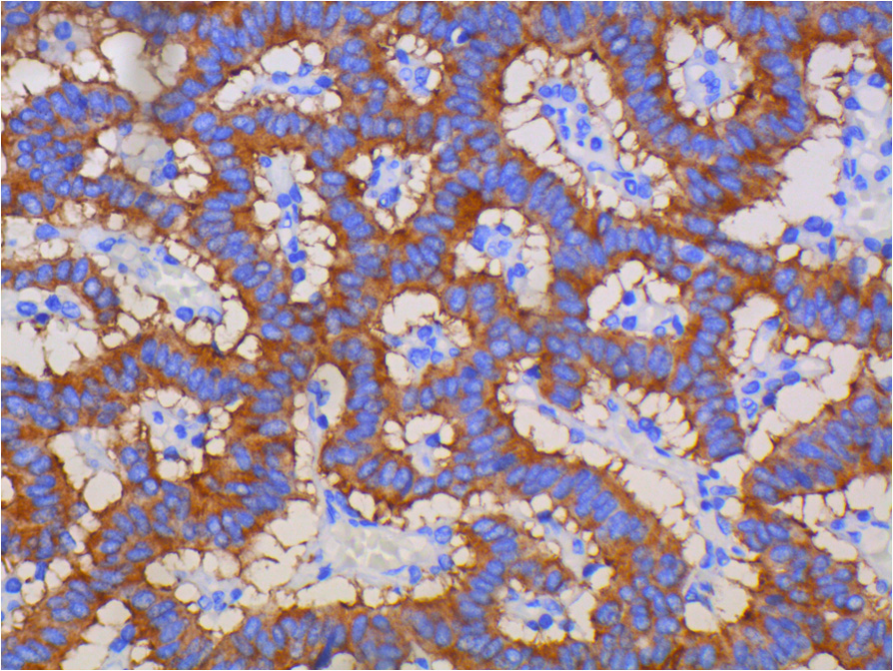
Intense cytoplasmic reactivity for synaptophysin (×400)

The mass was measured 2.8 cm × 2.8 cm × 2.5 cm in size, was well-defined. The patient had no other abnormal findings, such as suspected distant metastasis or lymph node metastasis. Radical nephrectomy was performed on 14/9/2005. On gross examination, the nephrectomy specimen measured 11.5 cm × 6.5 cm × 4.5 cm and the tumor was a solid, grayish-brown mass measured 3.5 cm × 2.5 cm × 2.2 cm in size. Histologic examination demonstrated trabecular and ribbonlike patterns with minimal fibrotic stroma. The cytoplasm was granular and eosinophilic with uniform round to oval nuclei with finely stippled chromatin and inconspicuous nucleoli. Mitoses were not found (0 per 10 high-power fields) on H&E stain. The final pathologic examination revealed a well-differentiated neuroendocrine (carcinoid) tumor confined to the kidney (Fig. 1). Immunohistochemical stains demonstrated labeling with chromogranin, neuron-specific enolase and synaptophysin (Fig. 2), and the proliferation index was less than 2 % as measured by immunohistochemistry for Ki-67. Further radiation and chemotherapy were not received.

**Fig. 3:**
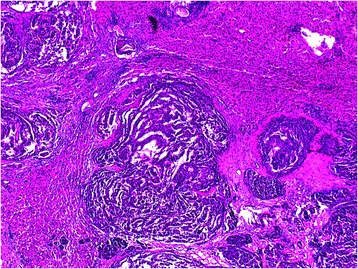
Renal carcinoid tumor with liver metastasis, tumor cells are arranged in trabecular and glandlike appearance (H&E × 200)

**Fig. 4:**
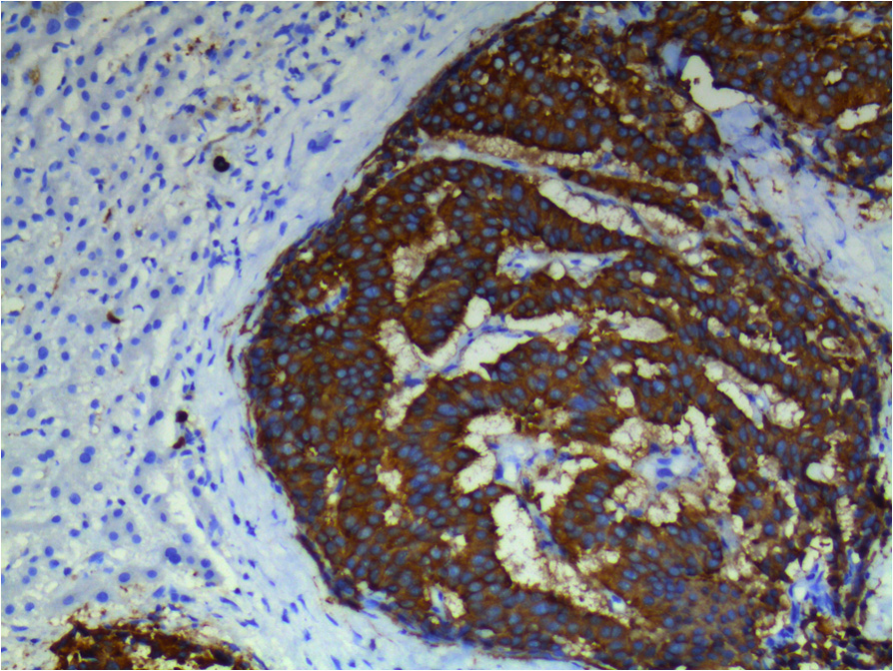
Intense cytoplasmic reactivity for synaptophysin (×400)

Nine years after radical nephrectomy, computed tomography of the abdomen demonstrated a 2 cm × 1.8 cm cyst mass in the right liver and multiple high density shadow in gallbladder. No enlarged lymph nodes were found in the abdominal cavity. Cholecystectomy and extirpation for hepatic cyst were performed by laparoscopy surgery. Histologic examination demonstrated trabecular and glandlike growth (Fig. 3), moderate and uniform nuclei and mitoses weren't found (0 per 10 high-power fields). Advanced immunohistochemistry revealed that the lesion was positive for synaptophysin (Fig. 4) and CD56, but negative for chromogranin and neuron-specific enolase. These features are supported in a neuroendocrine tumor. Due to the similar pathologic characteristics between the primary renal carcinoid tumor and liver tumor. The final pathology report indicated a carcinoid tumor of the left kidney with liver metastasis.

## Discussions

Carcinoid tumors occur most commonly in the gastrointestinal tract and respiratory tract. Primary renal carcinoid tumors are exceedingly rare and since the first reported case in 1966, less than 100 cases have been reported in the literature. Due to a small number of cases, biological behavior of these tumors is undetermined. Although the majority of patients in previous reports were diagnosed with either local or distant metastatic disease at the time of initial diagnosis, most patients were asymptomatic and demonstrated prolonged survival. The most common site for metastatic is regional lymph nodes, other locations include liver [1, 5], bone [6, 7] and lung [1]. The previous literature report liver metastatic disease at the time of surgery, only two patients developed liver metastases within 5 and 6 months of surgery [1, 4]. This is the rare case reported liver metastasis followed up after radical nephrectomy for 9 years.

The cell of origin of renal carcinoid tumors has not been determined. It seems that the tumor is not arise in the kidney and perhaps originate from scattered neuroendocrine cells derived from acquired or congenital abnormalities, because no neuroendocrine cell was detected within the renal parenchyma or hilum [8] and renal carcinoid tumors showed absence of reactivity with PAX-2 and PAX-8 [9]. The two markers have been shown to be associated with the developing mesonephric tissue and may serve as immunohistochemical markers of renal tumors. The predominant histologic pattern of the tumor includes tightly packed cords with minimal stroma, trabecular growth with prominent stroma, focal solid nests of cell or focal glandular lumina, other more rare histopathologic features were calcification and lymphocytic infiltrates. In our case, both kidney and liver lesion histologically show trabecular and glandlike appearance, moderate and uniform nuclei with finely stippled chromatin, but the later lack ribbon patterns. Mitoses and necrosis weren’t found.

Immunohistochemical stains suggest that neuroendocrine markers synaptophysin, chromogranin and CD56 were positive in both lesions, TTF-1, WT-1 and CDX-2 were negative [10]. It’s interesting that immunostaining of chromogranin was at least focal positive in kidney lesion, but negative in liver lesion. We are not well understood. It is speculated that protein expression of chromogranin and neuron-specific enolase was changed during the period of liver metastasis. It should be noticed that the relatively low specificity of neuron-specific enolase as a neural marker, because neuron-specific enolase immunostaining is observed in the normal kidney at the level of distal/medullary tubules, and in a high percentage of renal cell neoplasms [11].

The differential diagnosis of renal carcinoid tumor includes small cell carcinoma, primitive neuroectodermal tumor(PNET), neuroblastoma, paraganglioma, metanephric adenomas. In contrast to small cell carcinoma, renal carcinoid tumors lack a brisk mitotic rate, apoptotic activity, nuclear molding, necrosis and high proliferation indices. Furthermore, most small cell carcinomas lack the typical organized architecture that is distinctive for the carcinoid tumor [12]. Both PNET and renal carcinoid tumor can demonstrate solid areas and regions of rosettelike structures, although unlike renal carcinoids, PENT is strong immunoreactivity for CD99 and have the t(11; 22)(q24; q12) translocation with the fusion transcript between the EWSR1 gene(22q12) and the FLI gene(11q24) [13]. Paragangliomas and neuroblastomas arising in kidneys are exceedingly rare, although both can mimic renal carcinoid. Paragangliomas more commonly contain a nested pattern of neuroendocrine cells with granular basophilic cytoplasm surrounded by S100 positive sustentacular cells. Neuroblastoma contain Homer-Wright rosettes, neurofibrillary stroma which were not found in renal carcinoid tumors. Metanephric adenomas and renal carcinoid tumors are both composed of tightly packed small, monotonous cells with uniform nuclei and inconspicuous nucleoli. In contrast to renal carcinoid tumor, metanephric adenomas have round acini with embrynal appearance and psammoma bodies were commonly seen. Furthermore, metanephric adenomas lack immunoreactivity for neuroendocrine markers such as synaptophysin, chromogranin, and CD56.

Radical nephrectomy is the gold standard treatment for renal carcinoid, partial nephrectomy is a good alternative regarding the location and diameter of the tumor. Other neo/adjuvant treatment, such as chemotherapy, sandostatin, targeted therapy, radiotherapy or local lymph node dissection, but no trial has shown its direct impact on survival.

Neuroendocrine tumors (NET) commonly express serum somatostatin receptors as determined using somatostatin receptor scintigraphy with radiolabeled form of somatostatin analog octreotide. Octreotide not only plays an important role in decreasing the symptoms of hormonal excess but is considered a first-line antineoplastic systemic therapy for patients with a positive octreoscan [14]. Octreotide may have activity against primary or metastatic renal carcinoid [15]. In our case, octreotide have not been used postoperatively. There need more clinical trials to evaluate the effect of octreotide.

The prognosis of renal carcinoid tumors is not predictable because of their rarity. The majority of cases did not present evidence of disease after treatment. According to 2010 WHO classification and grading system, 6 previously reported cases of renal carcinoid tumor should be classified as renal neuroendocrine tumor grade 2 (mitotic activity 2–20/10 high-power fields and/or 3–20 % Ki-67 index level) [1, 15, 16]. These patients seem to be with a worse prognosis, because 5 of 6 renal NET grade 2 cases previously reported had metastasis and 2 of 6 patients died from disease.

## Conclusions

Renal carcinoid tumors exhibit heterogenous behavior. Although it seems that no good prognostic factors to predict the outcome of patients, Ki-67 index level and mitotic rate might be useful to aid pathologists and clinicians in trying to predict the outcome of a similar case.

## Consent

Written informed consent was obtained from the patient for publication of this case report and any accompanying images. A copy of the written consent is available for review by the Editor-in-Chief of this journal.
